# Comparative Analysis of Manual Dexterity of Dental Students at Ajman University following One Academic Year of Preclinical Training Sessions: A Longitudinal Cohort Study

**DOI:** 10.1055/s-0042-1758793

**Published:** 2022-12-19

**Authors:** Musab Saeed, Mohammed B.Q. Alfarra, Mawada Hassan Abdelmagied, Karrar Hadi, Tareq Aljafarawi, Noor Al-Rawi, Asmaa T. Uthman, Salem Abu Fanas, Natheer H. Al Rawi

**Affiliations:** 1 Department of Clinical Sciences, College of Dentistry, Ajman University, Ajman, United Arab Emirates; 2 Centre of Medical and Bio-allied Health Sciences Research, Ajman University, Ajman, United Arab Emirates; 3 Department of Basic Sciences, College of Dentistry, Ajman University, Ajman, United Arab Emirates; 4 Department of Pharmaceutical Sciences, College of Pharmacy, University of Sharjah, Sharjah, United Arab Emirates; 5 Department of Diagnostic and Surgical Dental Sciences, College of Dentistry, Gulf Medical University, Ajman, United Arab Emirates; 6 Department of Oral and Craniofacial Health Sciences, College of Dental Medicine, University of Sharjah, Sharjah, United Arab Emirates

**Keywords:** preclinical training, manual dexterity, dental students, Purdue Pegboard Test, O'Connor Tweezer Dexterity Test

## Abstract

**Objectives** Dental students must complete two stages of training, namely, preclinical training on phantom head models and clinical training on actual patients to acquire the practical skills required by their Bachelor of Dental Surgery program.

Our objectives are to evaluate the level of improvement of the manual skills obtained by third-year dental students after one full academic year of preclinical training courses using dexterity tests under direct and indirect vision and to compare the improvement among male and female dental students under the same conditions.

**Materials and Methods** A total of 72 preclinical students participated in our cohort trial, each of whom was assigned a random identification number that was only known to the researchers. After the beginning of the academic year, the experiment was performed under identical conditions for both the O'Connor Tweezer Dexterity Test and the Purdue Pegboard Test. The examinations were conducted at two distinct times: T0 before phantom laboratory training (the beginning of preclinical sessions) and T1 after phantom laboratory training (9 months after T0).

**Statistical Analysis** Signed-rank test of Wilcoxon over two separate periods (T0 and T1), comparisons were made between the direct and indirect visual dexterity test scores. In addition, the Mann–Whitney *U* test was used to compare results across gender. The statistical significance ( *p* -value) was set at below 0.05 with a confidence level of 95%.

**Results** A statistically significant difference was detected between the T0 and T1 assessments on the Purdue Pegboard Test and the O'Connor Tweezer Dexterity Test for all selected dentistry students in both direct and indirect conditions ( *p* < 0.001).

**Conclusion** Further investigation in other dental departments or schools, particularly those with different entry standards, is required prior to making a definitive conclusion about the use of these dexterity assessments as predictors of prospective dental students' performance.

## Introduction

Dentistry out of most professions requires the highest levels of both theoretical and practical knowledge. 1 Even if a student has a very high intelligence quotient, if lacks the necessary practical skills, he may have a restricted opportunity in the field of dentistry as a chosen career, since dentistry as a profession is considered to be limited and constrained. 2 However, the majority of dental schools focus on academic and cognitive qualities only in their admission criteria. Dental students at Ajman University acquire the essential manual skills in two stages: first, via preclinical simulated clinical activities, and second through clinical activities on actual patients. Therefore, the preclinical sessions are of high importance in preparing and equipping the dental students with the necessary manual skills that would enable them to join the second phase of training. However, some sorts of dexterity assessment for the dental students after the first phase of training would be quite beneficial for gauging the effectiveness of the preclinical phase. Manual dexterity is the ability to accomplish a job utilizing fingers, hands, and arms. Hand dexterity plays an important role in performing daily activities, work-related functions, and leisure activities. The hand must be able to do jobs demanding both great strength and extremely delicate and sensitive motions. 3

Students need to obtain complementing theoretical and practical instruction to progress to a standard stage in their education. In this situation, manual dexterity plays a significant role in the change from theory to practice. Competencies like spatial intelligence and fine motor abilities are frequently acquired in preclinical training laboratories. 4 5

Manual dexterity is exemplified by the capacity to use tools, grasp and manipulate small objects, and coordinate minute, precise motions. To safeguard the patients' well-being, you must also conduct these tasks safely. In the field of occupational therapy, manual dexterity tests are typically used to evaluate fine motor impairments in wounded patients and to determine a healthy person's adaptation to specialized fine assembly occupations. Using the occupational therapy manual dexterity evaluation, several studies have been conducted over the past 80 years to examine the connection between fine motor abilities and the competency required to meet preclinical dentistry criteria. 6

The Purdue Pegboard Test, the Minnesota Manual Dexterity Test, the Box and Block Test, the O'Connor Dexterity Tests, and the Functional Dexterity Test are the most often used tests to measure manual dexterity. Even though each of these tests measures dexterity, they are all different from one another and are therefore suggested for various objectives. 7 8

Given the importance of enhancing manual dexterity in dental training programs, it is proposed that a set of tests relevant to the subject be chosen to aid in the learning process. Hence, our objectives are to evaluate the level of improvement of manual skills obtained by third-year dental students after one full academic year of preclinical training courses using dexterity tests under direct and indirect vision and to compare the improvement among male and female dental students under the same conditions.

## Materials and Methods

Prior to the commencement of data collection, the College Research Ethics Committee authorized our longitudinal cohort project. On February 21, 2021, we received ethical permission from the Ajman University Research Ethics Committee; the approval number is D-H-F-2020-Dec-6. After describing the purpose of the experiment and emphasizing the need of data confidentiality, we obtained the participants' informed consent.

In the current investigation, all relevant data were utilized to calculate the sample size needed to produce statistically sound information from which conclusions may be drawn about the entire population. All third-year dental students from Ajman University's College of Dentistry were included in the research population. Moreover, a specific age range was determined which is 20 to 25 years old. Students with premedical courses outside the university, transfer students, and students with any form of psychomotor problems were excluded.

The sample size ( *n* ) was computed using an online OpenEpi link using the Kish method for sample size estimate with a significance level of 95%, a margin of error of 5%, and a response rate of 50%. The minimum representative sample size was 70.

Seventy-two preclinical students participated in the present cohort study. Each participant was randomly allocated an identifying number known only to the researchers to ensure anonymity. Students list for each section was obtained and systematic random sampling was performed using *k=N/n* in which *k* is the systematic sampling interval, *N* is population size, and *n* is the sample size. One researcher was allocated for each test assessment. At the end of the school year, the experiment was performed under identical conditions for both the Purdue Pegboard Test and the O'Connor Tweezer Dexterity Test.

**Fig. 1:**
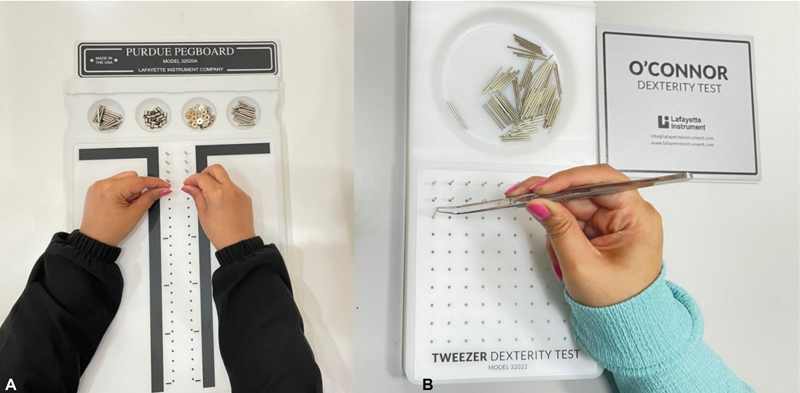
( **A** ) Purdue Pegboard Test using direct vision. ( **B** ) O'Connor Tweezer Dexterity Test using direct vision.

*Purdue Pegboard Test* : This test is a board with two columns of holes. The distal portion of the board has four cups: a cup with washers, a cup with collars, and two cups on the right and left with 25 pins each. The assessment consists of four problems. For the first three tasks, as many pins as possible must be placed into holes on the circuit board within 30 seconds, and for the assembly task, within 60 seconds. The score for each of the four tasks is determined by the number of pins placed. The roles vary in the following ways: First utilizing the dominant hand and then the nondominant hand, the third assignment was completed using both hands by gripping one pin in each hand and simultaneously putting them into two holes ( Fig. 1 ).

The Purdue direct assembly task was then completed. During assembly, the participant uses both hands constantly to assemble four components, beginning with a pin, threading a washer and a collar, and ending with a washer. After completing the four tasks with direct vision, the subjects performed the same four tasks using indirect vision.

*The O'Connor Tweezer Test of Dexterity* : This challenge is comprised of a 100-hole board and a 100-pin cup. The participant places each of the 100 pins using tweezers and their dominant hand. Throughout this examination, O'Connor Tweezers for direct and indirect vision were utilized. The time necessary to place each pin in a hole was set at 5 minutes, and the score was determined accordingly ( Fig. 1 ).

**Fig. 2:**
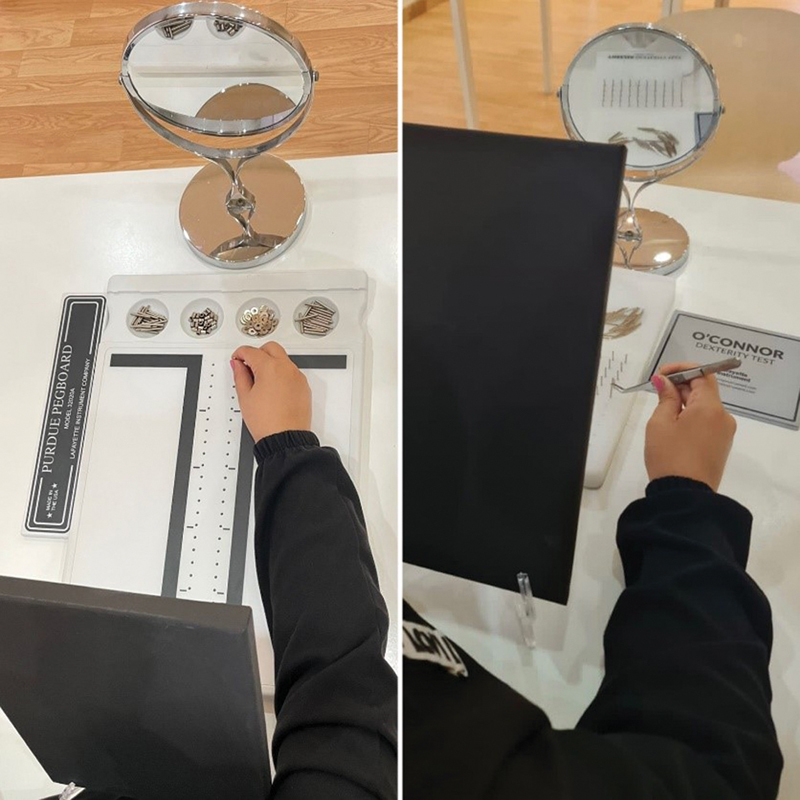
Purdue Pegboard Test and O'Connor Tweezer Dexterity Test using indirect vision.

To achieve indirect vision, the test board is covered with a blackboard shield, preventing participants from seeing the board directly and allowing them to complete the tasks using a mirror. To eliminate distractions, all students took the exam in a quiet, peaceful, and well-kept setting. Before beginning each level, each participant got the same, clear instructions from a single instructor through written notes. Students were free to stretch their legs by walking around the classroom between tasks ( Fig. 2 ).

Experiments were conducted at two time points: T0: before to phantom training (beginning of preclinical sessions), and T1: following phantom training (9 months after T0).

### Statistical Analysis

The collected data were tabulated, and statistical analyses were conducted using SPSS 28.0 (SPSS, Inc, Chicago, Illinois, United States).

In the present cohort, intraclass correlation coefficient analysis was done to confirm the consistency of the measurement at different times, and reliability was evaluated and reported accordingly.

The collected data were compiled and summarized as descriptive findings in terms of mean and standard deviation for all test scores received from each student. The data were not normally distributed, as shown by the Shapiro–Wilk test, hence nonparametric tests were employed. Signed-rank test of Wilcoxon over two separate periods (T0 and T1), comparisons were made between the direct and indirect visual dexterity test scores. In addition, the Mann–Whitney *U* test was used to compare results across gender.

Spearman's correlation was utilized to examine the strength of each test's effect at T0 and T1 across all individuals. Similarly, a linear regression analysis was utilized to estimate the degree of progress across genders linked with the same level of dental education using manual dexterity tests. In the current examination, the statistical significance ( *p* -value) was set at less than 0.05 with a confidence level of 95%.

## Results

There were a total of 72 third-year students who volunteered to engage in this evaluation, with more than half (65.28%) of the participants being females (34.72%).

Test-retest reliability was calculated using a two-way random model, O'Connor Tweezer, employing the intraclass correlation coefficient across several measurements at various timing (T0 and T1). The direct and indirect tests demonstrated good to excellent reliability (0.78 and 0.95, respectively), but the Purdue test indicated a reliability level ranging from 0.017 (bad dependability) to 0.669 (moderate reliability) ( Table 1 ). Spearman's correlation analysis was utilized to examine the strength of the relationship between the degree of progress in both direct and indirect vision and the preclinical sessions. There was a strong positive correlation between the Purdue direct assembly task ( *r* = 0.770), the O'Connor Tweezer Direct (0.716), and the O'Connor Tweezer Indirect ( *r* = 0.864), with a significant difference ( *p* < 0.001) indicating that both direct and indirect manual skills improved significantly over time.

**Table 1:** Two-way random effects model where both people effects and measures effects are random

Intraclass correlation coefficient							
	Intraclass correlation	95% confidence interval		* F* -test with true value 0			
		Lower bound	Upper bound	Value	df1	df2	Significance
Purdue direct using dominant hand	0.669	0.472	0.793	3.025	71	71	< 0.001
Purdue direct using nondominant hand	0.589	0.343	0.743	2.433	71	71	< 0.001
Purdue direct using both hands	0.392	0.028	0.619	1.644	71	71	0.019
Purdue direct assembly task	0.017	–0.571	0.385	1.017	71	71	0.471
Purdue indirect using dominant hand	0.375	0.001	0.609	1.600	71	71	0.025
Purdue indirect using nondominant hand	0.255	–0.191	0.534	1.342	71	71	0.109
Purdue indirect using both hands	0.631	0.411	0.769	2.714	71	71	< 0.001
Purdue indirect assembly task	0.407	0.053	0.629	1.688	71	71	0.014
O'Connor Tweezer Direct	0.785	0.657	0.866	4.658	71	71	< 0.001
O'Connor Tweezer Indirect	0.959	0.935	0.975	24.671	71	71	0.000

Abbreviation: df, degrees of freedom.

On the other hand, a weak positive correlation was observed between students who completed Purdue Indirect with their dominant and nondominant hands ( *r* = 0.111 and *r* = 0.208, respectively) with no significant difference ( *p* = 0.35, *p* = 0.079). Similarly, a moderate positive correlation was observed between students' use of their dominant and nondominant hands with Purdue Direct ( *r* = 0.397 and *r* = 0.364, respectively) but with a significant difference ( *p* 0.001 and *p* 0.002, respectively) ( Table 2 ).

**Table 2:** Spearman's correlation analysis between students at T0 and T1

Variables	Correlation coefficient	Significance (two-tailed)	95% confidence intervals	
			Lower	Upper
Purdue direct using dominant hand	0.397	< 0.001	0.176	0.581
Purdue direct using nondominant hand	0.364	0.002	0.138	0.554
Purdue direct using both hands	0.256	0.030	0.018	0.466
Purdue direct assembly task	0.770	< 0.001	0.651	0.852
Purdue indirect using dominant hand	0.111	0.355	–0.131	0.340
Purdue indirect using nondominant hand	0.208	0.079	–0.032	0.425
Purdue indirect using both hands	0.589	< 0.001	0.408	0.726
Purdue indirect assembly task	0.427	< 0.001	0.210	0.604
O'Connor Tweezer Direct	0.716	< 0.001	0.575	0.815
O'Connor Tweezer Indirect	0.864	< 0.001	0.788	0.914

**Fig. 3:**
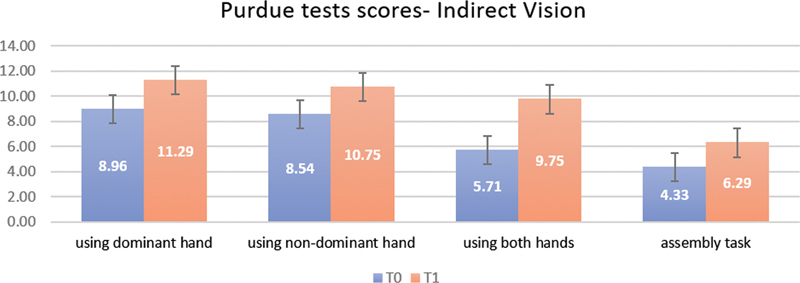
Purdue Pegboard Test indirect vision at T0 and T1.

Fortunately, the overall mean of all the tests showed a significant difference when comparing the scores of T0 and T1 for all dental students; the level of improvement of the Purdue direct test using the dominant hand increased from 14.75 ± 2.62 at T0 to 15.71 ± 1.89 at T1; further analysis revealed a significant level of improvement for the indirect Purdue test using the dominant hand ( *p* < 0.001), from 8.96 ± 1.38 to 11.29 ± 2.35 ( Fig. 3 ).

Initially, the O'Connor Tweezer Test direct vision average score was 80.17 ± 13.91 (at T0) out of 100 during a 5-minute time limit and improved to 92.50 ± 9.90 at the end of the preclinical sessions; similarly, the indirect vision average score improved from 13.25 ± 9.26 (at T0) out of 100 during a 5-minute time limit to 21.13 ± 10.73 at the end of the preclinical sessions, with a significant difference ( Table 3 ).

**Table 3:** Comparison between the participant's skills at the beginning and at the end of the preclinical courses

		Mean	Standard deviation	Standard error	Minimum	Maximum	Test statistics	*p* -Value
Purdue direct using dominant hand	T0	14.75	2.62	0.309	10.00	21.00	–2.973	0.003
	T1	15.71	1.89	0.223	12.00	21.00		
Purdue direct using nondominant hand	T0	13.58	2.15	0.254	9.00	17.00	–3.290	0.001
	T1	14.58	1.90	0.224	10.00	21.00		
Purdue direct using both hands	T0	11.42	2.19	0.258	8.00	18.00	–2.944	0.003
	T1	12.17	2.39	0.282	10.00	19.00		
Purdue direct assembly task	T0	7.38	1.86	0.219	3.00	10.00	–4.768	< 0.001
	T1	8.83	1.22	0.144	6.00	10.00		
Purdue indirect using dominant hand	T0	8.96	1.38	0.162	6.00	12.00	–5.720	< 0.001
	T1	11.29	2.35	0.277	5.00	14.00		
Purdue indirect using nondominant hand	T0	8.54	1.27	0.149	6.00	11.00	–5.929	< 0.001
	T1	10.75	2.08	0.245	6.00	14.00		
Purdue indirect using both hands	T0	5.71	1.25	0.147	4.00	9.00	–7.082	< 0.001
	T1	9.75	2.73	0.322	4.00	14.00		
Purdue indirect assembly task	T0	4.33	1.26	0.148	2.00	7.00	–6.402	< 0.001
	T1	6.29	1.98	0.234	3.00	12.00		
O'Connor Tweezer Direct	T0	80.17	13.91	1.640	52.00	100.00	–6.452	< 0.001
	T1	92.50	9.90	1.167	67.00	100.00		
O'Connor Tweezer Indirect	T0	13.25	9.26	1.092	1.00	33.00	–7.083	< 0.001
	T1	21.13	10.73	1.265	7.00	46.00		

**Fig. 4:**
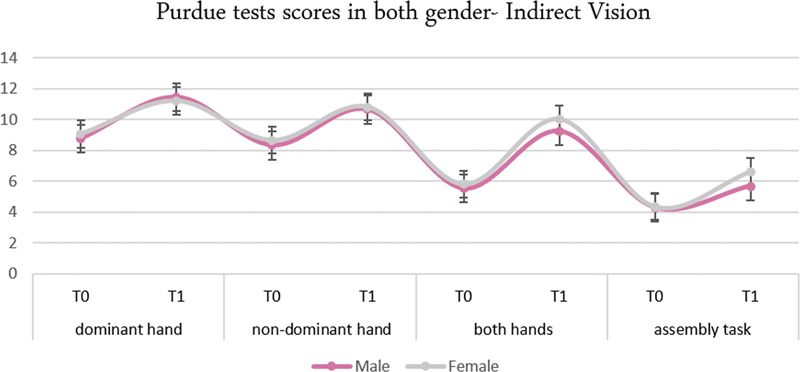
Purdue Pegboard Test indirect vision at T0 and T1 in both genders.

**Fig. 5:**
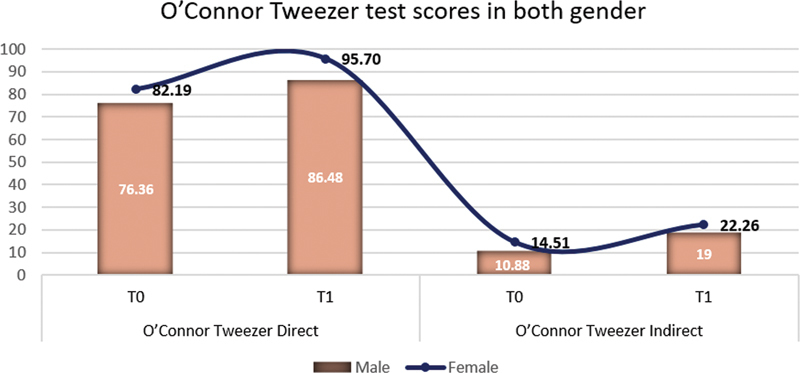
O'Connor Tweezer Test direct and indirect vision at T0 and T1 in both genders.

Male and female students did not differ significantly in terms of the degree of improvement using nearly all of the selected measures of assessments for direct and indirect vision ( Fig. 4 ), with female students reporting slightly higher scores (22.26 ± 11.20) than male students (19.00 ± 9.66) when using O'Connor Tweezer indirectly, but this difference was not statistically significant ( *p* = 0.337). However, when the O'Connor Tweezer Test was performed using direct vision, females scored considerably higher ( *p* < 0.001) than males (95.70 ± 6.27 vs. 86.48 ± 12.53) ( Table 4 and Fig. 5 ).

**Table 4:** Comparison in relation to gender at the beginning and at the end of the preclinical courses

		Male ( *N* = 25)		Female ( *N* = 47)			
		Mean	Standard deviation	Mean	Standard deviation	Test statistics	*p* -Value
Purdue direct using dominant hand	T0	13.52	2.58	15.40	2.42	–2.760	0.006
	T1	15.64	2.08	15.74	1.81	–.239	0.811
Purdue direct using nondominant hand	T0	13.20	2.27	13.79	2.08	–0.951	0.341
	T1	14.56	1.73	14.60	2.01	–0.622	0.534
Purdue direct using both hands	T0	10.84	1.89	11.72	2.30	–1.554	0.120
	T1	12.40	2.50	12.04	2.35	–.702	0.483
Purdue direct assembly task	T0	7.52	1.39	7.30	2.07	–0.073	0.942
	T1	8.60	1.38	8.96	1.12	–1.054	0.292
Purdue indirect using dominant hand	T0	8.76	1.48	9.06	1.33	–0.771	0.441
	T1	11.44	2.43	11.21	2.33	–0.869	0.385
Purdue indirect using nondominant hand	T0	8.32	1.14	8.66	1.32	–1.148	0.251
	T1	10.64	2.64	10.81	1.74	–0.309	0.757
Purdue indirect using both hands	T0	5.52	1.36	5.81	1.19	–0.954	0.340
	T1	9.24	3.00	10.02	2.57	–0.967	0.333
Purdue indirect assembly task	T0	4.28	0.89	4.36	1.42	–0.333	0.739
	T1	5.68	1.70	6.62	2.06	–1.819	0.069
O'Connor Tweezer Direct	T0	76.36	15.19	82.19	12.90	–1.571	0.116
	T1	86.48	12.53	95.70	6.27	–3.418	< 0.001
O'Connor Tweezer Indirect	T0	10.88	8.41	14.51	9.53	–1.477	0.140
	T1	19.00	9.66	22.26	11.20	–0.961	0.337

In the linear regression analysis, gender was utilized as the independent variable coefficient ( *β* ) and test score values were used as the constant ( *α* ). The prediction equation displayed a weak association between each independent variable and dependent variable, with the exception of the O'Connor Tweezer Test direct vision, which was positively and independently related to the test score ( *β* = 0.447) and considered a good predictor of test scores. Other measurements displayed a range of weak positive (Purdue test using dominant hand directly *β* = 0.026) and weak negative regression analysis (Purdue test using both hands directly *β* = –0.02672) ( Table 5 ).

**Table 5:** Linear regression: predictors: (constant), gender

Variable	* R* ^2^	Adjusted *R* ^2^	Standardized coefficients beta	Significance
Purdue direct using dominant hand	0.001	–0.014	0.026	0.825
Purdue indirect using dominant hand	0.002	–0.012	–0.046	0.699
Purdue direct using both hands	0.005	–0.009	–0.072	0.550
Purdue indirect using both hands	0.019	0.005	0.137	0.251
O'Connor Tweezer Direct	0.199	0.188	0.447	< 0.001
O'Connor Tweezer Indirect	0.021	0.007	0.145	0.223

## Discussion

The Johnson O'Connor Research Foundation defines tweezer dexterity as “the ability to operate with small instruments and to do delicate activities such as dentistry, surgery, nursing, mechanical sketching, watchmaking and repair, and miniature instrument construction.” 9

Studies utilizing tweezer dexterity tests developed by the Johnson O'Connor Research Foundation have not established a high manual dexterity aptitude for dentists. However, Weinstein et al discovered a negative correlation between high dexterity scores on the Johnson O'Connor Tweezer Dexterity Test and peer evaluation of restorative work quality for practicing dentists. 1

According to a 1992 paper by Simon and Chambers, the Johnson O'Connor Tweezer Dexterity Test, which is believed to test both speed and accuracy, was used in their search for a profile of aptitudes defining effective dentists. 10 This study likewise failed to substantiate the conventional perspective, reporting a mean score for dentists on tweezer dexterity that was not significantly different from the average for the general population. 9

In our study, test-retest reliability was determined using the intraclass correlation coefficient across multiple measurements at different timing (T0 and T1), and both the direct and indirect O'Connor Tweezer tests demonstrated good to excellent reliability, with coefficients of 0.785 and 0.959, respectively. In another study, repeatability was determined by individually assessing the first and second halves of the O'Connor Tweezer Test. This strategy yielded a 0.7925 repeat correlation coefficient. 9 The Johnson O'Connor Research Foundation claims a dependability of 0.91. 11

The current study also revealed that completion of dental school practical courses has a substantial effect on tweezer dexterity. The tweezer dexterity and pegboard test scores of third-year students at the end of the academic year differed considerably.

The vast majority of researches demonstrate that manual dexterity is a necessary dental skill. According to one study, dexterity is the cornerstone of these abilities. 12 However, the outcomes of the research evaluating the importance of gaining these skills before enrolling in dentistry school are inconsistent. Numerous studies link low psychomotor exam performance to a lack of engagement in these activities prior to dentistry school, emphasizing the significance of hobbies such as jewelry making and other manual dexterity enhancing activities. 13 Due to the rigorous curriculum of dentistry school, it is strongly advised that students improve their dexterity before enrollment to better adapt to manual procedures. 14

To execute dental procedures, a dentist must be able to operate with precision in a rather limited space. The specific manual dexterity requirements for the dentistry profession are still unknown. The usefulness of manual, stereopsis, hand-eye coordination, cognitive, perceptual, handwriting, and drawing skills in dentistry is a subject of research. 15 16 17

Despite the fact that dental school admission procedures are normally successful, a small percentage of students each year are unable to acquire restorative dentistry manual skills. These students have difficulty completing the curriculum and fail to fulfill course requirements in preclinical courses. 18 19 As these students do not learn these tasks at an acceptable level and as a result, their clinical skills are defined as substandard; therefore, the University of California, San Francisco School of Dentistry proposed to determine if manual dexterity assessments were correlated with subsequent grades in preclinical training and faculty perceptions of acceptable performance in skills that indicate that students are ready to advance to the clinic and such tests might be a useful addition to the dental admissions process. 19

This study demonstrated that overall mean of Purdue and O'Connor dexterity tests showed a significant difference when comparing the scores of T0 to T1 for all dental students, the level of improvement of Purdue using dominant hand increased from 14.75 ± 2.62 at T0 to 15.71 ± 1.89 at T1 for the direct vision and from 8.96 ± 1.38 to 11.29 ± 2.35 for the indirect vision, these results showed that performing both Purdue and O'Connor dexterity tests is appreciably more difficult under indirect vision. Students had worse scores under indirect vision than direct vision. Furthermore, compared with the Purdue test, the discrepancies between the direct and indirect vision conditions for the O'Connor Tweezer Test were far more pronounced.

O'Connor Tweezer Test direct vision average score was 80.17 ± 13.91 (at T0) out of 100 during a 5-minute time limit and improved to 92.50 ± 9.90 at the end of the preclinical training sessions; similar behavior was reported for the indirect vision but at lower scores, in which average score was 13.25 ± 9.26 (at T0) out of 100 during a 5-minute time limit and improved to 21.13 ± 10.73 at the end of the preclinical training sessions, with a significant difference ( *p* < 0.001). Similarly, Lugassy et al observed that the performance of students on the Purdue and O'Connor examinations considerably improved following phantom training (T1) under direct and indirect vision settings. 6

It is assumed that the Purdue test, which involves the fingertips and lasts 30 to 60 seconds, is less relevant to daily dental clinical practice than the O'Connor test, which involves the use of tweezers and is significantly more time consuming; therefore, the time for the O'Connor test was limited to 5 minutes.

Assembly is the most difficult task on the Purdue tests, requiring both cognitive and mental efforts. 6 It is hypothesized that during their studies, dental students engage in a more cognitive and mental practice than dentists, and hence do better on this test. 20

In long-term dental procedures, tweezers (O'Connor test) are utilized more often than fingers (Purdue test) for rapid, short-term jobs. As described in the literature, the O'Connor test can thus predict the manual dexterity abilities required for clinical practice. 4 Consistent with the findings of earlier investigations, we found that the ability to use indirect vision is a preclinical skill. 21 However, dentists continue to encounter frustration throughout their careers when attempting to employ indirect vision, and therefore avoid utilizing dental mirrors for the maxillary teeth. 21

One of the reliable hypotheses in literature stated that occupational therapy tests 9 22 would be sensitive to the improvement of students' manual skills after training in the preclinical course. Higher scores were found at T1 versus T0. Positive transfer occurs when the practice of one motor activity leads in a discernible improvement in the performance of another task. Positive transmission is a controversial subject among scholars. It was shown that this transfer relied on identical components shared by two performances 23 24 (i.e., for transfer to occur, the two performances should be as similar as possible). Others, however, argue that motor abilities are particular, which means that a person's performance on one movement test is not always indicative of their performance on another. 25 Therefore, training with simple, approachable instruments such as the Purdue and O'Connor tasks under indirect vision conditions can significantly improve these skills. Our findings are consistent with occupational therapy research indicating that practice increases performance. 26 In contrast to the O'Connor test, the Purdue exams are extremely quick and do not truly reflect the extended concentration required by a dentist, particularly in indirect vision. In our study, we found a weak correlation between gender and level of improvement, as both sexes improved significantly. Among the tests, however, the O'Connor Tweezer Test for direct vision favored females, implying that females are relatively quick learners, which is consistent with Lugassy et al's observation. 6 Although there is no statistically significant difference between males and females, the results demonstrated that females performed somewhat better than males on activities requiring fine motor abilities, confirming the findings of Upadhayay and Guragain. 27 However, we determined that manual dexterity may be greatly enhanced with training over time, which concurs with Luck et al's findings in their study. 28

Although we observed a significant improvement in the skills of females performing the direct vision O'Connor Test, indicating that females are quick learners, there is no literature regarding the difference in the learning speed of motor skills between the sexes, and this remains an area for future research.

Peters et al concluded in 1990 that people with larger fingers had a more difficult time picking up the thin pegs than those with smaller fingers. This was the case with the majority of the males as compared with the females in performing the Purdue tests, despite the fact that we were testing the level of improvement rather than who is superior, and this may explain why the females scored slightly higher on finger-based tests. 29

According to Giuliani et al, the manual dexterity of students who finished the whole phantom training course increased significantly. This result is consistent with studies by Luck et al 28 and Gansky et al 30 which concluded that manual dexterity can be acquired and improved through exercise, and that ability tests should primarily be used to identify the weakest students prior to preclinical courses to provide them with additional training so they can achieve the required dental performances. 31

As proven by research, females perform differently during different phases of menstruation. Multiple papers provide qualified support for the hypothesis that the high levels of gonadal steroids present during the luteal phase of the menstrual cycle may facilitate skills favoring females, but hinder skills favoring males. 32

A substantial positive correlation was found in relation to the Purdue direct assembly task ( *r* = 0.770), O'Connor Tweezer Direct (0.716), and O'Connor Tweezer Indirect ( *r* = 0.864) with a significant difference ( *p* < 0.001) in which a significant improvement was realized over a period of time in both direct and indirect manual skills. Students in their third year retook these tests 9 months later, and while this may be considered a retake, there is a substantial confounding variable that may have altered the results: extensive practice in the phantom course. As a result of the major gains in the dexterity tests, it is projected that correlations would diminish. The study had several limitations, including the fact that all individuals came from the same dentistry school and that no correlations were assessed between manual dexterity assessments and final grades in the phantom course. Before a definitive decision can be made regarding the use of these manual dexterity tests with distinct eye directions as potential predictors of success in preclinical studies, extensive research in other dental faculties or schools, particularly those with different admission requirements, is required. 6

Furthermore, occupational therapy manual dexterity assessments have been used in several studies over the past 80 years to look at the relationship between fine motor abilities and the aptitude needed to pass preclinical dental courses. Block carving, 30 the tremometer test, 28 the two-hand coordination machine, 28 the O'Connor Tweezer Dexterity Test, 9 the Purdue Pegboard Exam, 9 and other assessments are reported in literature. 33 34 However, the greatest predictive test, is the subject of disagreement. 17 While de Andrés et al 35 claimed that this test predicts poor student performance and identifies low-performing pupils in advance, Lundergan et al 9 concluded that the O'Connor test has little predictive capacity. An additional screening instrument for dentistry students might be utilized, according to a different study, which revealed that a stainless steel mouth-simulation frame with two plastic arches and 32 holes was more relevant to dental activities. 6 The wire-bending test has been shown to be an extra and useful method for selecting applicants for dentistry schools reported by the University of Hamburg in Germany. 36

We can find a positive prediction for females using linear regression, and we can anticipate that girls will do better with time, but males will not. The prediction equation revealed a weak relationship between each independent variable and dependent variable, with the exception of the O'Connor Tweezer Test direct vision, which was positively and independently related to the test score and regarded as a good predictor of test scores. Other measurements demonstrated a range of weak positive correlations.

A logistic regression as was the case with Lugassy et al, was not able to predict the amount of success as compared with the students' grades since we did not include the students' grades and opted to take the level of improvement as the success. 4

## Conclusion

Despite the fact that manual dexterity vastly improves with preclinical manual training, some sort of practical tests that can be assessed objectively should be added to dental school admission criteria to predict the degree of improvement that may be observed after preclinical training. Future studies will continue to focus on how gender variations affect how students learn new skills or improve their motor skills.
